# Facile Synthesis and Reuse of Magnetic Black Carbon Magnetite (BC-Mag) for Fast Carbamazepine Removal from Water

**DOI:** 10.3390/nano10020213

**Published:** 2020-01-26

**Authors:** Nan Cai, Philip Larese-Casanova

**Affiliations:** 1 Guangdong Provincial Key Laboratory of Emergency Test for Dangerous Chemicals, Guangdong Engineering and Technology Research Center of Online Monitoring for Water Environmental Pollution, Guangdong Institute of Analysis, Guangzhou 510070, China; cainan@fenxi.com.cn; 2 Department of Civil & Environmental Engineering, Northeastern University, 360 Huntington Avenue, Boston, MA 02115, USA

**Keywords:** graphene, black carbon magnetite, sorption processes, freundlich isotherm, water treatment

## Abstract

Magnetic carbonaceous nanomaterials are needed in water treatment applications because they can offer both carbon surfaces for sorption of organic pollutants and ease of material magnetic retrieval for regeneration and reuse. In this study, we employed a facile one-step method to synthesize a black carbon-magnetite composite (BC-Mag) by high-temperature annealing of black carbon and hematite. The nanocomposite was easily dispersed and stable in water owing to the presence of negatively charged oxygen surface functional groups. Sorption kinetics with dissolved carbamazepine showed a rapid initial uptake with equilibrium achieved within only minutes. The sorption extent can be described with the Freundlich model, and surface area normalized sorption affinity was an order of magnitude greater than conventional granular activated carbon. The sorption extent of neutral carbamazepine remained constant between pH 2–10 while surface zeta potential decreased. BC-Mag can be reused for the sorption of carbamazepine up to six times without significant loss of the sorption extent.

## 1. Introduction

Water and wastewater treatment strategies often employ adsorption technologies for the removal of organic, inorganic, and metallic pollutants [1,2,3]. Carbonaceous sorbents hold strong affinities for organic micropollutants owing to their strong abilities to form hydrophobic and aromatic interactions [4,5,6,7]. Recent advances in carbonaceous sorbents have been to incorporate nanotechnology features and fine-tuning of carbon surfaces for enhanced reactivity [8,9]. Nanometer-sized features of carbon sorbents can provide significantly high specific surface areas and chemically tunable surfaces (aromatic carbon Csp^2^, aliphatic carbon Csp^3^, or functional groups) available for contaminant sorption [10,11]. The graphene structure in the form of carbon nanotubes and graphene nanosheets has shown particularly superior performance compared to activated carbon for the removal of organic micropollutants [12,13]. More disordered forms of carbon that have been evaluated as alternative sorbents include biochar, coal, and soot [14,15].

Nanosized amorphous carbon has also been formed on surfaces of magnetic iron oxide nanoparticles for manipulation within test waters using a magnetic field [16]. These particles typically contain a core-shell structure where the exposed carbon shell provides available sorption sites and the protected iron oxide core (typically magnetite, Fe_3_O_4_) is sensitive to applied magnetic fields [11,17,18]. This combination of physical properties allows retrieval of the nanoparticles for regeneration and reuse [19,20]. Carbon-coated magnetic iron oxides are typically formed within hydrothermal conditions where dissolved organic molecules (e.g., glucose) become charred upon pre-formed nanomagnetite particles [21]. The result is typically a coating of amorphous carbon (as opposed to ordered graphite-like sheets) upon 10–50 nm-sized iron oxide cores. Repeated washing of the particles with alcohol and water is required to remove unbounded charred compounds, and so far allowable mass production is low due to low concentrations of nanoparticles produced and extensive washing.

The purpose of this paper is to evaluate magnetic black carbon nanoparticles as a sorbent for the removal of trace organic micropollutants in water. Black carbon is nanometer-sized spherical particles of pure carbon (typically <20 nm) commercially available in powdered form. These nanoparticles typically hold very high surface areas that, if exposed to the solution phase, could provide surface sites for rapid and extensive sorption of organic molecules. Black carbon was chosen because it is one of the smallest nanosized carbons and, in order to maximize its exposed surface area, it was chosen to deposit it on the outer surfaces of magnetite particles. In contrast, other magnetite-carbon composites using carbon nanotubes (CNTs) [22,23,24] or a char [25] have been synthesized for water treatment applications, but these featured the magnetic particles decorated on top of the carbon surfaces where both carbon and magnetite can participate in sorption processes. The nanosized black carbon spheres might also have their surface area more readily exposed compared to activated carbon where much of the surface area resides in pore structures, which limits sorption rates. While black carbon and magnetite composites have not yet been evaluated well for water treatment applications, they have been examined for their properties as absorbents for carbon dioxide [26,27,28] and as microwave absorbing composites [29]. Synthesis of black carbon attached to magnetic iron minerals has been reported to occur around 1000 °C, and higher temperatures can lead to dissolution of carbon within the iron oxide and reprecipitation as an amorphous exterior coating upon cooling [30]. The synthesis of black carbon magnetic particles is suited for product yields of several grams after processing within tube furnaces, and no post-washing is required due to the annealing of loosely-bound organic molecules. This synthesis route may prove to be less resource- or time-intensive compared to hydrothermal synthesis.

Graphene, carbon nanotube, black carbon and other traditional carbonaceous sorbents have high sorption performance to remove most contaminants from water systems, however, all those materials may be difficult to be retrieved and reused in stirred reactors. One way to make the particles retrievable is to synthesize magnetic graphitic nanoparticles in that way the used particles could be easily retrieved by magnet and regenerated by organic solvents. Chandra et al. developed magnetite-graphene hybrids via a chemical reaction, and the results showed that the composites can remove arsenic nearly completely within the parts per billion (ppb) level [31]. Zhu et al. reported a facile thermo-decomposition process to synthesize magnetic graphene nanocomposites for fast chromium removal [32]. 

Carbamazepine, a widely used anticonvulsant for the treatment of epilepsy, is one of the 11 most frequently detected endocrine disrupting compounds (EDCs) in water systems [33]. It is marginally soluble in water (~200 mg L^−1^) which allows the study of a range of concentrations, and lets recalcitrant be removed. Carbamazepine can remain untransformed and pass through all conventional water treatment processes [34,35] and advanced processes such as membrane bioreactors [36]. Advanced oxidation processes (AOP) can be used to break down and transform carbamazepine [37,38]; however, the formation of unknown byproducts has been a concern. Therefore, sorption processes and techniques using amorphous carbon-based nanomaterials could be employed to produce favorable carbamazepine removal efficiency. Carbamazepine, with two aromatic rings, is expected to interact strongly with the π electron system of black carbon. 

This work employed a facile method to synthesize a novel carbonaceous nanomaterial, black carbon magnetite (BC-Mag), which fuses black carbon to magnetite surfaces to enable the sorbents magnetic and easy to retrieve. BC-Mag’s structure, particle sizes, surface area, charge and chemistry were characterized by various instrumentations. Sorption rates and sorption capacity were evaluated with carbamazepine by black carbon-magnetite composite (BC-Mag) under different buffer conditions, and sorption mechanisms were discussed. Surface charge and effect of pH on BC-Mag were also tested over the pH range 2 to 12. The recovery of carbamazepine and the regeneration of BC-Mag were evaluated for practical considerations.

## 2. Materials and Methods 

### 2.1. Material Synthesis and Characterization

Three grams of hematite and 1.45 g of black carbon powders were used as the starting materials for the synthesis of BC-Mag. The powders were placed within a 50 mL polypropylene tube and vigorously vortexed for 30 min, and then rotated end-to-end for 24 h to achieve a good mixing. The mixture was heated at 1000 °C in a tube furnace at heating rate of 3 °C per minute, and kept for 2 h at temperature within a nitrogen atmosphere to exclude oxygen which can oxidize the black carbon surface. This ratio of hematite to black carbon and synthesis temperature was chosen because it was sufficient to reduce Fe(III) in hematite to Fe(II) in magnetite instead of full reduction to Fe(0) [30], and abundant Fe(0) was to be avoided to minimize problems with potential Fe(0) corrosion products. BC-Mag was characterized for physical and chemical properties prior to use. Specific surface area (SSA) was measured by five-point Brunauer-Emmett-Teller (BET) analysis (80 °C degassing temperature) with a Quantachrome Nova 2200e instrument (Ashland, VA, USA). The surface carbon–oxygen and iron–oxygen functional groups were identified by X-ray photoelectron spectroscopy (XPS) using a Surface Science Instruments X-probe SSX-100 (Cornell Center for Materials Research Shared Facilities). Samples were mounted on carbon tape, and spectra were analyzed with CasaXPS software and fit with Gaussian-Lorentzian peak shapes after a Shirley background correction. Transmission electron microscopy (TEM) was performed with a Jeol JEM-1010 instrument (Peabody, MA, USA), and scanning electron microscopy (SEM) images were obtained with a Hitachi S-4800 instrument (Santa Clara, CA, USA) (NEU Electron Microscopy Core Facilities). X-ray Diffraction (XRD) was performed with a Rigaku UltimaIV instrument with CuKα radiation (Wilmington, MA, USA). Zeta potential was measured using a Malvern Nanosizer ZS90 (Westborough, MA, USA) for dilute aqueous suspensions of 0.1 g L^−1^ BC-Mag in 5 mM NaCl with pH modified using 0.1 M HCl or 0.1 M NaOH. Particle sizes of BC-Mag components were estimated by measurement of TEM images using ImageJ software (n = 300 for black carbon particles, and n = 125 for magnetite particles). In-solution particle sizes of entire composites were measured by dynamic light scattering (DLS) using a Brookhaven 90Plus particle size analyzer (Holtsville, NY, USA) in solutions similar to zeta potential measurements.

### 2.2. Sorption Experiments

The sorption experiments were conducted in batch reactors with carbamazepine and BC-Mag in buffer solution. The buffer solution was prepared by 20 mM NaCl and 1 mM NaHCO_3_ in deionized water (>18 MΩ cm) with small additions of 0.1 M HCl or 0.1 M NaOH for pH adjustment. The stock solutions of carbamazepine were prepared in methanol. In order to minimize cosolvent effects, the spike volumes of carbamazepine stock solutions were kept below 0.1% of reactor total volumes. After carbamazepine was spiked to buffer solution, initial samples were taken into 2-mL high pressure liquid chromatography (HPLC) vials and sealed. The sorption reaction was initiated by addition of the sorbent BC-Mag and immediately agitated by rotator disk. To separate the solid phase of BC-Mag and the aqueous phase of the solution and stop the reaction, a strong magnet was first used to retain the nanoparticles within the reactor and prevent them from clogging the syringe-tip filters. Samples were then placed into HPLC vials by filtering through 0.2 micron PTFE syringe-tip filters and stored at 4 °C prior to analysis. The sorbed carbamazepine amount was calculated by time point concentrations minus initial concentrations and normalized to mass of BC-Mag added.

Kinetic experiments were conducted in 100 mL glass bottles with an initial carbamazepine concentration of 90 mg L^−1^ and a BC-Mag concentration of 0.5 g L^−1^.The solution pH was pH 7.2, and the solution was stirred rapidly with a bar magnet. Isotherm experiments were carried out within 30 mL glass vials with PTFE-l--ined septa with 5–200 mg L^−1^ carbamazepine and 1.0 g L^−1^ BC-Mag at pH 7.2. These glass vials were rotated end-over-end for mixing with a contact time of 20 min. The solution pH remained at 7.2 ± 0.2 in the experiment. The pH edge experiment was conducted in a pH range of 2.0–12.0 with initial carbamazepine concentrations of 80 mg L^−1^ and BC-Mag concentrations of 0.5 g L^−1^ for 20 min. All experiments were conducted at room temperature (25 °C).

### 2.3. Desorption and Regeneration

Recovery experiments were performed with repeated retrievals of BC-Mag and washings and further use. BC-Mag was first exposed to carbamazepine at 10 mg L^−1^ in 10 mL of buffer solution within 30 mL glass vials for 20 min. After reaction, BC-Mag solids were pulled by the magnet to reactor a sidewall within 5 min. 1 mL of supernatant was carefully sampled with 0.2 micron PTFE syringe-tip filters, and the other 9 mL of supernatant was discarded and replaced with 10 mL ethanol (95+% pure, anhydrous, denatured with up to 5% *v*/*v* ether, Acros) as a desorbant to promote recovery of carbamazepine and regeneration of BC-Mag owing to carbamazepine’s higher solubility in ethanol (0.108 mol/L) compared to water (0.001 mol/L) [39]. The suspension was quickly shaken and vortexed for 30 s and then placed on rotator disk for 5 min. Solids were separated using magnet, and the supernatant was sampled using 0.2 micron PTFE syringe-tip filters for HPLC measurement of carbamazepine recovery. After the removal of the supernatant of ethanol, the damp BC-Mag powders were heated at 50 °C in the oven for 30 min until dried. A reuse cycle started by the injection of 10 mL fresh 10 mg L^−1^ carbamazepine stock solution into the glass vial with dried BC-Mag, and vigorously vortex the suspension for 30 s. Then the glass vial was placed on the rotator disk for 20 min contact time, and the sorption and desorption steps were repeated for a total of five regeneration steps. Sorbed carbamazepine mass was calculated by difference in concentrations before and after each step, and carbamazepine recovery percentage was calculated through dividing the measured carbamazepine concentration in ethanol by the sorbed carbamazepine mass. The sorption percentage of carbamazepine in every step was calculated by dividing the change in concentrations before and after each step with the initial concentrations.

### 2.4. Quantification of Carbamazepine

Carbamazepine concentrations were quantified by HPLC (Agilent 1260 Infinity Quaternary LC, Santa Clara, CA, USA) with an ultraviolet light (UV) detector using a 4.6 × 50 mm ODS Hypersil C18 column (Thermo Scientific) with injection volume of 10 µL, 40% acetonitrile and 60% HPLC grade water as eluent, flow rate of 1 mL/min, and wavelength of UV absorbance of 220 nm.

## 3. Results and Discussion

### 3.1. Characterization of Black Carbon Magnetite

**Figure 1:**
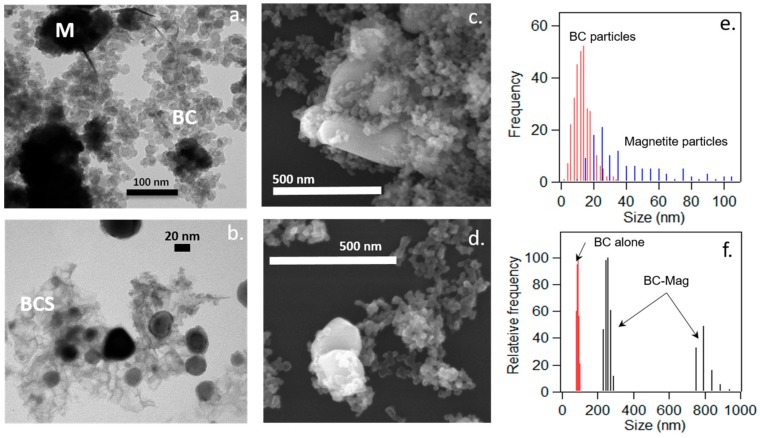
Transmission electron microscopy (TEM) images (**a**,**b**), scanning electron microscopy (SEM) images (**c**,**d**), individual component size distributions (**e**), and dynamic light scattering (DLS) whole particle size distributions (**f**) of black carbon-magnetite composite (BC-Mag) particles synthesized at 1000 °C. In the TEM images, electron-dense regions are the iron minerals (M), lighter spherical particles are black carbon (BC), and lighter sheet-like structures are transformed black carbon (BCS). In the SEM images, the magnetite appears as the larger, brighter phases.

The heat treatment of hematite and black carbon at 1000 °C resulted in a fusion of the particles and a phase transformation of the iron oxides. TEM images revealed the presence of black carbon particles accumulated near surfaces of the iron minerals (Figure 1). The black carbon particles took the appearance of either fused primary particles or fused but more crumpled sheet-like (which were more rare). The black carbon primary particles had an average size of 12 ± 5 nm with a fairly narrow size distribution and smaller than most of the embedded iron minerals which had an average size of 42 ± 33 nm and a broader size distribution. BC-Mag when suspended in solution had an effective diameter of 457 ± 13 nm as measured by DLS, but the size distributions reveal most of the composite particles to have sizes in the 240–270 nm range and a smaller population at 750–840 nm. All BC-Mag particles were larger than untransformed BC aggregates (80–100 nm) in solution, indicating BC fusion to larger sizes did occur. SEM images revealed a bulbous morphology of the whole aggregates, and the iron minerals are partly covered by fused black carbon particles. The heat treatment did not significantly transform the black carbon particles in size or identity; such transformation (e.g., dissolution of carbon into the iron oxides, volatilization) usually occurs at higher temperatures [30].

**Figure 2:**
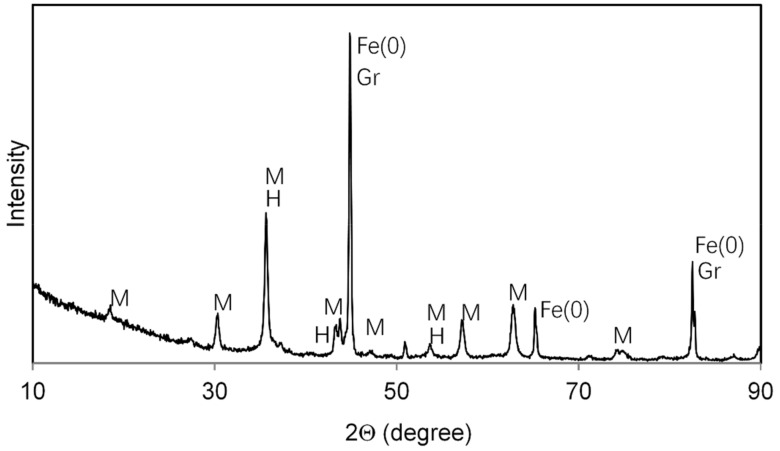
X-ray Diffraction (XRD) pattern of BC-Mag particles synthesized at 1000 °C. M = magnetite, H = hematite, Fe(0) = iron metal (alpha form), Gr = graphite.

The high temperature reaction did result in transformation of most of the parent hematite into a mixture of more reduced iron minerals including magnetite (Fe_3_O_4_) and zero-valent iron (Fe(0)), as revealed in an XRD pattern (Figure 2). Reflections were compared to locations for mineral standards as provided in standard powder diffraction file cards. The reduction of Fe(III) in hematite to Fe(II) in magnetite and Fe(0) was possible at high temperature with elemental carbon as an electron source and without oxygen in the gas stream. Because some hematite reflections overlap with magnetite, it is possible some hematite was not transformed fully. The black carbon is mostly graphitic in nature, as indicated by the graphite reflections present in the XRD pattern. It is possible some of the magnetite contains maghemite (γ-Fe_2_O_3_) because standard reflections of these minerals are indistinguishable. The BC-Mag particles were easily retrievable from aqueous suspension using a strong magnet, and this observation supports the identification of magnetite and Fe(0) owing to their significantly larger magnetic susceptibility values compared to that of hematite, which is not attracted to magnets. The measured SSA was 125 m^2^ g^−1^, which is higher than some graphene oxides (15–74 m^2^ g^−1^) and carbon nanotubes (CNTs) (112 m^2^ g^−1^) but lower than high surface area graphene oxide (771 m^2^ g^−1^) and granular activated carbon (GAC) (1181 m^2^ g^−1^) used in our previous study [40]. One reason for BC-Mag’s lower SSA is due to the iron and iron oxide’s higher density than carbonaceous materials.

**Figure 3:**
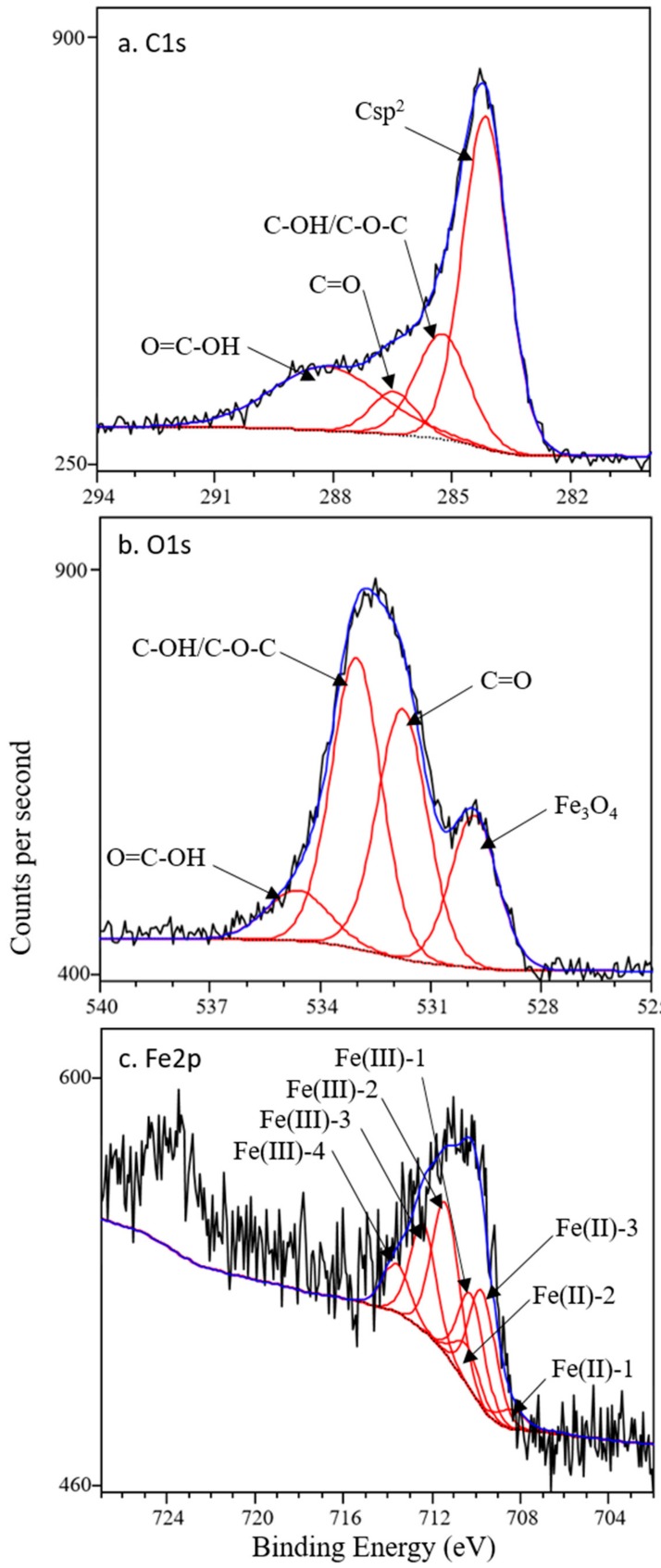
XPS spectra of BC-Mag particles, (**a**) C1s, (**b**) O1s, (**c**) Fe2p. Black lines are measured data, red lines are modeled peaks, and blue lines are overall model fit.

**Figure 4:**
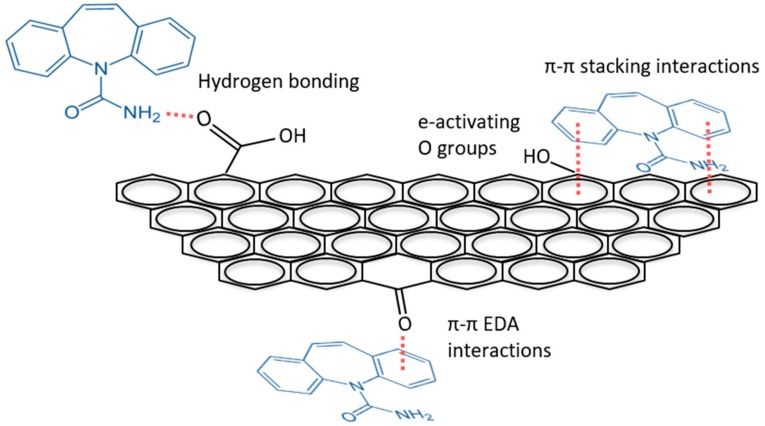
Schematic of the likely sorption mechanisms of carbamazepine on the functionalized graphitic surfaces of BC-Mag.

Surface chemistry was characterized with high resolution XPS scans (Figure 3). Carbon sp^2^ was the most prevalent form of carbon observed, owing to the conjugated π system of black carbon, and accounts for the graphite detection in the XRD pattern. Oxygen-containing surface functional groups were identified in both C1s and O1s spectra and included the C–O bonds (as hydroxyl C–OH or ether C–O–C), carbonyl groups (C=O), and carboxyl groups (O=C–OH). Model fits (Table 1) are consistent with prior models for black carbon [41,42,43]. While π−π stacking could be the predominating physisorption mechanism between carbamazepine and BC-Mag, the surface O groups could also contribute to binding. For one, the exposed O groups could form hydrogen bonds with the amine group on carbamazepine. The hydrogen bonding mechanism was previously observed to contribute to the binding of larger ring-structured biomolecules to nanosized graphene oxide [44] Carbonyl groups on carbon sorbents have been proposed to participate in π−π electron-donor acceptor interactions [4,40] The C=O groups identified on BC-Mag could serve as π electron donor groups with carbamazepine acting as the π acceptor, caused by the amide group having an electron withdrawing effect on carbamazepine’s ring structures. As electron-activating groups, the phenolic and ether surface groups could also influence the π−π binding mechanism by increasing π electron density of BC-Mag Csp^2^ rings which could increase strength or extent of sorption [40,45]. A schematic of these four sorption mechanisms between BC-Mag and carbamazepine is illustrated in Figure 4.

Magnetite was identified in O1s and Fe2p spectra, but no evidence of any Fe(0) (expected at 706.5 eV) was present in Fe2p spectra, which suggests any formed Fe(0) resided below the iron oxide surface. Only the Fe2p_3/2_ feature was modeled due to the low signal, and magnetite was the sole iron oxide phase considered due to its positive identification with XRD. The seven-peak model of Fe^3+^ and Fe^2+^ multiplets of Grosvenor et al. [46] was sufficient to describe the data, and this model suggested a Fe^3+^:Fe^2+^ ratio of 2.4:1 which is slightly under stoichiometric with respect to Fe^2+^ (expected 2:1).

### 3.2. Sorption Rates and Capacity

**Figure 5:**
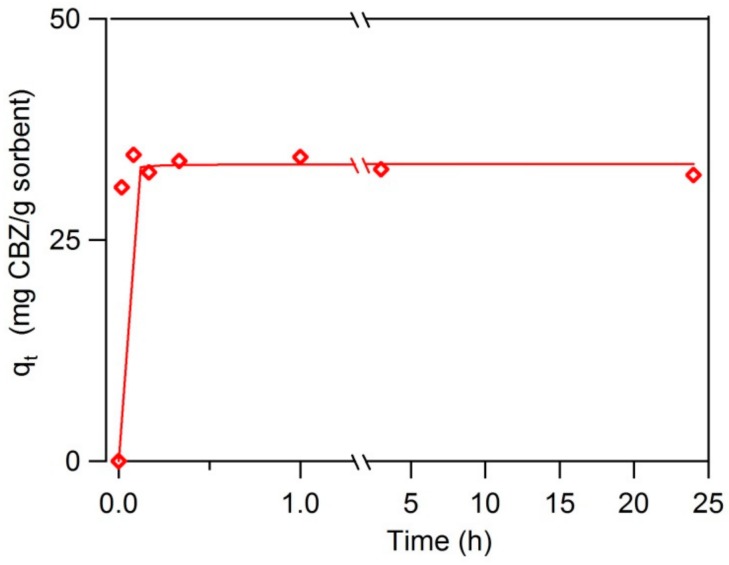
Time profile of carbamazepine sorption on BC-Mag 0.5 g L^−1^, solid lines are pseudo-second order kinetic model simulation.

The kinetic data of carbamazepine sorption on black carbon magnetite shows a very fast uptake at the beginning and a quick process to reach the equilibrium (Figure 5). The pseudo-second order rate law best describes carbamazepine sorption on BC-Mag with correlation coefficient *R^2^* > 0.999. The pseudo-second order kinetic model is *dq_t_/dt = k_2_(q_e_ – q_t_)^2^*, where *q_t_* (mg g^−1^) is sorbed carbamazepine concentration on BC-Mag at time *t* (h), *q_e_* is sorbed carbamazepine concentration on BC-Mag at equilibrium, and *k_2_* (g mg^−1^ h^−1^) is the pseudo-second order rate constant. The pseudo-second-order model assumes that sorption extent is controlled by the amount of open surface sites rather than the dissolved sorbate concentration, and that chemisorption is the rate controlling step. Sorption rates parameters are calculated as listed in Table 2, and include the fitted rate constant *k_2_*, the equilibrium sorption extent *q_e_*, and the calculated relaxation time *t_r_ = 1/(k_2_ q_e_)*, which represents the time required for surface sites to reach half-saturation. The fast sorption of carbamazepine on BC-Mag was reflected through the high *k_2_* value and small *t_r_* value which equals to only 4.3 seconds. This relaxation time is one of the smallest among other carbonaceous nanosorbents (1.4–68 s) and GAC (36 min) examined for carbamazepine adsorption at the same solids concentration (0.5 g L^−1^) [40].

This material may represent the fastest sorbent for organic pollutants in water such as the problematic EDCs, superior to most of other graphene and 500 times faster than traditional GAC. While the BC-Mag composite does have lower SSA than GAC and some graphene oxides previously tested, it is likely the SSA of BC-Mag is made artificially low due to the larger relative mass of the iron mineral component (BC alone can have SSA up to 1000 m^2^ g^−1^). The irregular, rounded nature of BC aggregates might have more exposed surfaces compared to flat graphene oxide, and GAC is known to have much of its surface area in pores which slows initial sorption kinetics. Therefore the very fast sorption kinetics is likely due to high surface area of the ~12 nm primary black carbon particles exposed in solution, which overcomes the mass limitation imposed by magnetite. Moreover, the equilibrium sorption extent on a mass sorbent basis here is on par with some graphene oxides under the same experimental conditions, but lower than multi-walled carbon nanotubes (MWCNTs) and GAC [40]. When sorption extent is normalized to surface area, BC-mag is similar to most graphene oxides and GAC, showing available surface area is a controlling factor to sorption extent, as expected.

**Figure 6:**
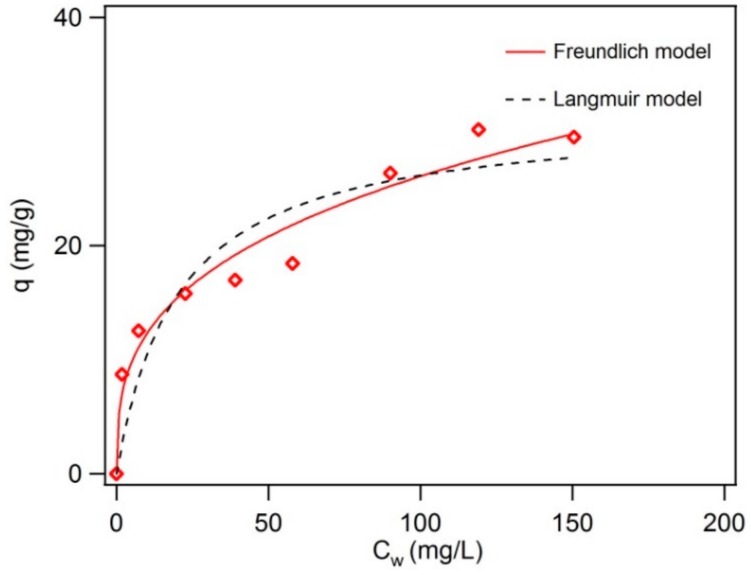
Sorption isotherms of carbamazepine on black carbon magnetite. Red solid line is Freundlich model simulation, and black dotted line is Langmuir model simulation. Experimental conditions: 1.0 g L^−1^ graphene, pH 7.2, 20 mM NaCl, 1 mM NaHCO_3_.

To better estimate sorption capacity, an isotherm study was performed, and the Freundlich model and Langmuir model were evaluated (Figure 6). The results showed that both of the two models can fit the data but the Freundlich model had a higher correlation coefficient *R^2^* of 0.95 compared to the *R^2^* of 0.82 for the Langmuir model (Table 3). This suggests that the heterogeneity in sorption sites exists rather than one defined site for carbamazepine on BC-Mag, which is reasonable because the various aromatic, aliphatic, and surface functional groups on black carbon, as identified by XPS, and potential O sites on magnetite should have different affinities for carbamazepine. By comparing to the sorption capacity of carbamazepine by different commercial graphene, MWCNTs, and GAC from our previous study [40], the sorption affinity *K_F_* is comparable to some graphene oxides but inferior to MWCNTs and graphene oxide with very high SSA (771 m^2^ g^−1^). This trend holds even when *K_F_* is normalized to SSA. However, *K_F_/SSA* for BC-Mag (0.05) is an order of magnitude greater than that for GAC (0.003) despite having much lower specific surface area. This is indicative of the surface sites of BC-Mag being far more accessible than the sites within the porous structure of GAC.

### 3.3. Effect of Solution pH

**Figure 7:**
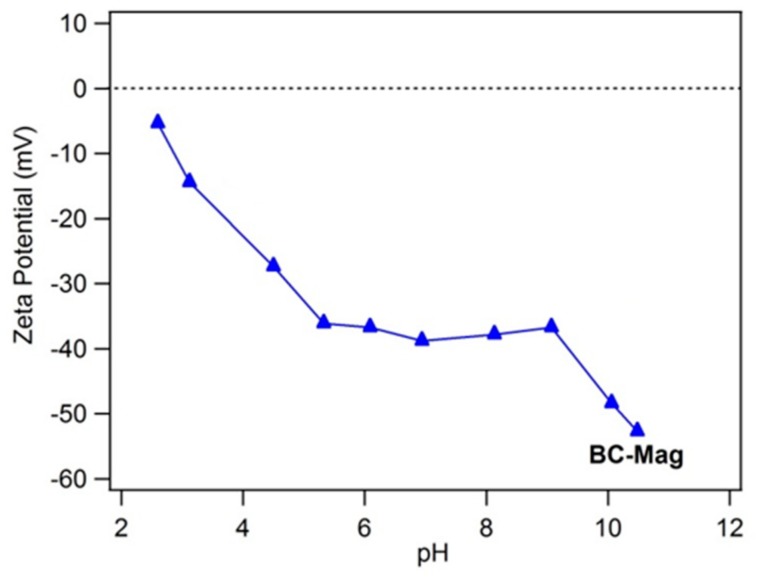
Zeta potential of black carbon magnetite. Experimental conditions for a: 0.1 g L^−1^ graphene in 5 mM NaCl.

Over the pH range 2 to 12, BC-Mag was always found to have negative zeta potential (Figure 7), which was even much more negative (e.g., −52.7 mV at pH 10.5) than what we observed for graphene oxide previously [40]. The negative charge was caused by deprotonation of surface functional groups such as the carboxyl (pK_a_ 4–5) and hydroxyl (pK_a_ ~10) groups as identified by XPS. The drastic decrease in zeta potential at pH > 9 is likely also due to deprotonation of surface O groups on magnetite which usually has an isoelectric point at pH 8–9 [47].

**Figure 8:**
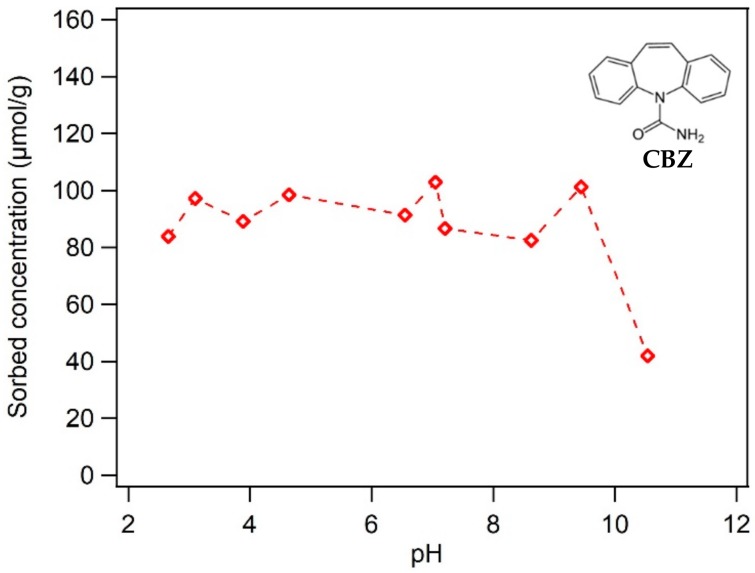
Effect of pH on the neutral sorbate carbamazepine. Experimental conditions: 0.5 g L^−1^ black carbon magnetite and initial sorbate concentrations of 70–80 mg L^−1^ in a buffer solution of 1 mM NaHCO_3_ and 20 mM NaCl.

In Figure 8, by varying pH from 2 to 10, the sorption of carbamazepine did not change because carbamazepine is a neutral compound with little electrical repulsion or attraction involved. However, at the more basic pH 10.5, sorption has been slightly decreased which might be due to increased electrostatic repulsion between the increasingly negative surface charge of BC-Mag and the partial negative charge of the amide O in carbamazepine. Nevertheless, the strongly negatively charged surfaces should be supportive of the sorption of cationic organic compounds.

### 3.4. Recovery of Carbamazepine and Regeneration of BC-Mag

**Figure 9:**
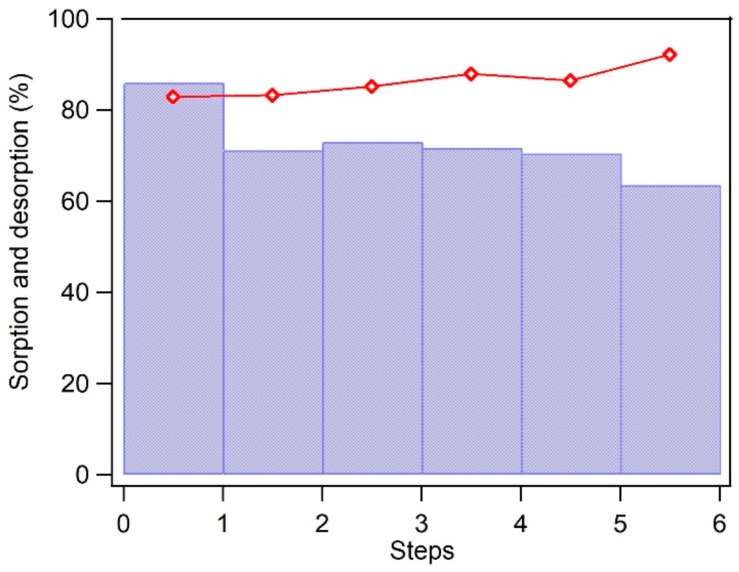
Carbamazepine sorption percentage by BC-Mag within consecutive six repeated steps of ethanol washing (bar graph) and the recovery of carbamazepine by ethanol as desorbant from every step (red dots and line). Experimental conditions: carbamazepine initial concentration 10 mg L^−1^, mass of BC-Mag 10 mg, solution volume 10 mL, buffer solution of 1 mM NaHCO_3_ and 20 mM NaCl.

To evaluate the stability and possibility of reuse of BC-Mag as sorbent in order to reduce the cost of sorption procedures and recovery of contaminants from wastewater, repeating applications for six steps of BC-Mag sorption experiments has been performed. The separation of solid phase and liquid phase in each step has been much easier resulting from the magnetism of synthesized magnetic sorbent BC-Mag with the help of a strong magnet alongside. Sorption efficiencies of carbamazepine on BC-Mag were shown in the bar graph of Figure 9. It was as high as 85% for the first run, then dropped to 70% and kept stable for the following four runs, and dropped to 65% at the last run. The decrease of removal efficiency is possibly due to a fraction of carbamazepine remained firmly bounded on the surface sites of BC-Mag and resisted to desorb even with ethanol washing. Also, it has been observed that during the process of BC-Mag separation from carbamazepine solution after sorption and washing processes with ethanol, a small portion of particles have been lost because they have weak or no magnetism and they could not be attracted by the magnet but stayed with the liquid. The efficiency of carbamazepine recovery was also presented in Figure 9 with red dots and line, and it remains stable at a high level of ca. 85%, which indicated ethanol as an effective solvent to extract carbamazepine. Also, a slight increase of carbamazepine recovery percentage may be due to desorption of the originally firmly bound molecules after six rounds of washing by ethanol.

## 4. Conclusions

The study demonstrated fast removal of carbamazepine with high capacity from water within minutes to reach sorption equilibrium by a black carbon-magnetite composite sorbent. Black carbon provides a high surface area of exposed surface sites for organic contaminant removal from water, and magnetite allows for easy recovery of sorbent in stirred reactor systems. The sorption extent of carbamazepine was hardly influenced by pH in the range of 2.0–10.0. Besides, the sorbed carbamazepine could be efficiently desorbed in ethanol with an average recovery percentage 85%, and the used BC-Mag could be easily separated from liquid solution with the aid of magnet and reused at least 6 times with removal efficiency higher than 65%. By comparing the sorption behavior of BC-Mag to commercial carbon-based sorbents such as GAC, CNTs, and graphene oxides with various SSA, BC-Mag has the advantage of having one of the fastest sorption kinetics based on relaxation time. With greater K_F_/SSA values than GAC, BC-Mag’s surface sites are far more accessible than GAC to sorb organic pollutants. Although CNTs and some graphene oxides have a greater sorption affinity than BC-Mag, BC could be a less expensive alternative carbon material. Finally, BC-Mag has the advantage of its magnetism allowing it to be easily retrieved from stirred suspension and reused.

In practice, BC-Mag can be an effective sorbent for carbamazepine removal from water, and the reuse and regeneration ability of BC-Mag could help to save cost in unit operations. Sorption capacity was comparable to some conventional carbonaceous sorbents, and it is possible capacity could be optimized by incorporating a higher amount of carbonaceous surfaces such as additional black carbon, graphene nanosheets, CNTs, or carbon dots, although the more specialized carbons might bring higher material costs. With black carbon being less expensive than high surface area CNTs and graphene oxides, and with BC-Mag synthesis requiring one curing step, BC-Mag is an attractive material for scaling up synthesis, and future work should investigate factors affecting scaled synthesis and their application to field water conditions. With a much faster sorption rate and higher sorption capacity comparing to conventional activated carbon, and the possible new properties of reusability, BC-Mag can serve as an alternative to conventional sorption materials in modern water treatment applications.

## Figures and Tables

**Table 1:** Model fits for X-ray photoelectron spectra of BC-Mag particles *^a^*.

	Binding Energy	Rel. Area	FWHM
	eV	%	
Cls			
C=C sp^2^	284.1	51.2	1.4
C–OH/C–O–C	285.2	18.8	1.5
C=O	286.5	6.5	1.3
O=C–OH	288.2	23.5	3.2
			
O1s			
Fe_3_O_4_	529.8	19.1	1.5
C=O	531.7	33.5	1.6
C–OH/C–O–C	533.0	39.0	1.6
O=C–OH	534.6	8.3	2
			
Fe2p_3/2_			
Fe(II)-1	708.4	3.3	1.4
Fe(II)-2	709.7	19.7	1.4
Fe(II)-3	710.4	6.6	1.4
Fe(III)-1	710.2	16.4	1.4
Fe(III)-2	711.3	27.9	1.5
Fe(III)-3	712.4	18.0	1.4
Fe(III)-4	713.6	8.1	1.4

*^a^* Binding energy = peak location on binding energy axis. Rel. area = percent relative area for each component. FWHM = full width at half maximum.

**Table 2:** Pseudo-second-order kinetic model parameters of carbamazepine sorption on BC-Mag. *R^2^* > 0.99 for all model simulations.

Sorbent	Sorbent Concentration g L^−1^	*t_r_* h	*k_2_* g mg^−1^ h^−1^	*q_e_* mg g^−1^	*q_e_/SSA* mg m^−2^
BC-Mag	0.5	0.0012	24.8	33.6	0.27

**Table 3:** Isotherm parameters for fitted Freundlich and Langmuir isotherm models of carbamazepine sorption on BC-Mag.

Freundlich Isotherm			Langmuir Isotherm		
*K_F_^a^*	*n*	*R^2^*	*K_L_* (L mg^−^^1^)	*q_max_* (mg g^−^^1^)	*R^2^*
5.8	0.33	0.95	0.05	31.8	0.82

^a^ All Freundlich modeling was performed on data with units of dissolved sorbate concentration in mg L^−1^ and sorbed sorbate concentration of mg g^−1^.

## Data Availability

Research data is available upon request.
